# Environmental Epigenetics Update and Boards

**DOI:** 10.1093/eep/dvz006

**Published:** 2019-05-14

**Authors:** Michael K Skinner

**Affiliations:** Center for Reproductive Biology, School of Biological Sciences, Washington State University, Pullman, WA 99164-4236, USA

*Environmental Epigenetics*, an Oxford University Press publication, just initiated its fifth year of operations with this Volume 5 Issue 1. We are a completely Open Access journal listed in PMC and PubMed, along with numerous other access sites. *Environmental Epigenetics* initiated its review to obtain an impact factor this year. Two special issues are planned this year in Epigenetics, Environment and Reproduction and in Epigenetic Transgenerational Inheritance, both associated with corresponding scientific meetings around the world. The amount and diversity of our published studies is increasing as the field of environmental epigenetics grows and expands. We are looking forward to another productive year and encourage you to consider submissions to *Environmental Epigenetics*.

The heart of the journal has always been the quality and breadth of the Environmental Epigenetics Editorial Boards. This Editorial is designed to extend a profound thanks to the Editorial Boards. From the beginning the Editorial Board, Consulting Editorial Board, and Editorial Review Board have provided advice and insights into the development and operations of the journal and its review process. I personally want to thank all those Editors that have acted as managing editors and reviewers. Without the dedication, experience and hard work of the Editorial Boards the journal would not exist. The current members of each of the Boards are listed below. You will see an outstanding group of dedicated and hardworking individuals that truly are the backbone of *Environmental Epigenetics*.

## Editorial Board

Baccarelli, Andrea

Chair and Leon Hess Professor

Environmental Health Sciences

Columbia University

Mailman School of Public Health

New York, NY, USA

Bales, Karen

Professor

Department of Psychology

University of California, Davis

Davis, CA, USA

Blumberg, Bruce

Professor

Department of Developmental and Cell Biology

University of California, Irvine

Irvine, CA, USA

Bonduriansky, Russell

Professor

Evolution & Ecology Research Centre

School of Biological, Earth and Environmental Sciences

University of New South Wales

Sydney, NSW, Australia

Chang, Howard

Virginia & D.K. Ludwig Professor of Cancer Genomics

Stanford University School of Medicine

Stanford, CA, USA

Cheng, Xiaodong

Professor

Molecular and Cellular Oncology Department

The University of Texas MD Anderson Cancer Center, USA

Dearden, Peter K.

Professor

Director of Genomics Aotearoa

Biochemistry Department

University of Otago

Dunedin, New Zealand

Dolinoy, Dana

Professor

University of Michigan

Environmental Health & Nutritional Sciences

Ann Arbor, MI, USA

Hoyo, Cathrine

Director

Epidemiology and Environmental Epigenomics Laboratory

North Carolina State University, USA

Kelly, William

Professor

Emory University

Department of Biology

O. Wayne Rollins Research Center

Atlanta, GA, USA

LaMerrill, Michelle

Associate Professor

Department of Environmental Toxicology

University of California at Davis

Davis, CA, USA

LaSalle, Janine

Professor

University of California, Davis

Davis, CA, USA

Mann, Melissa

Associate Professor

Obstetrics, Gynecology & Reproductive Sciences

Magee-Womens Research Institute

Pittsburgh, PA, USA

Mansuy, Isabelle

Professor

Lab of Neuroepigenetics

University/ETH Zürich

Brain Research Institute

Zürich, Switzerland

McCarrey, John

Professor

Department of Biology

University of Texas at San Antonio, USA

Meissner, Alexander

Associate Professor

Department of Stem Cell and Regenerative Biology

The Broad Institute

Harvard University

Cambridge, MA, USA

Metz, Gerlinde A.S.

Professor

Canadian Centre for Behavioural Neuroscience

Department of Neuroscience

University of Lethbridge

Lethbridge, AB, Canada

Osteen, Kevin

Professor

Vanderbilt University School of Medicine

Professor of Obstetrics and Gynecology

Nashville, TN, USA

Petronis, Art

Professor

University of Toronto

The Krembil Family Epigenetics Laboratory

Centre for Addiction and Mental Health

Toronto, ON, Canada

Rothstein, Mark

Founding Director of the Institute for Bioethics, Health Policy and Law

University of Louisville School of Medicine

Louisville, KY, USA

Shioda, Toshihiro

Associate Professor of Medicine

Harvard Medical School

Charlestown, MA, USA

Spencer, Hamish

Professor

Department of Zoology

University of Otago

Dunedin 9054, New Zealand

Sung, Sibum

Associate Professor

The University of Texas at Austin

Molecular Biosciences, College of Natural Sciences

Austin, TX, USA

Surani, Azim

Professor

The Gurdon Institute

University of Cambridge

Cambridge, UK

Szyf, Moshe

Professor

Department of Pharmacology

McGill University

Quebec, Canada

Waterland, Robert

Professor

Departments of Pediatrics and Molecular & Human Genetics

Baylor College of Medicine

USDA/ARS Children's Nutrition Research Center

Houston, TX, USA

Weitzman, Jonathan

Directeur du laboratoire

Université Paris Diderot

Center for Epigenetics

75205 Paris Cedex 13 France

Yan, Wei

Professor

Department of Physiology & Cell Biology

University of Nevada Reno School of Medicine

Reno, NV, USA

## Consulting Editorial Board

Gluckman, Peter

Professor

The University of Auckland New Zealand

GRAFTON

New Zealand

Hanson, Mark

Professor

University of South Hampton, UK

Jablonka, Eva

Cohn Institute

Tel Aviv University

Tel Aviv, Israël

Jégou, Bernard

Professor

INSERM (Institut National de la Santé et de la Recherche Médicale)

F-35042 Rennes CEDEX, France

Jirtle, Randy

Professor

Department of Biological Sciences

NC State University

Raleigh, NC, USA

Peterson, Richard

Professor

University of Wisconsin

Pharmaceutical Sciences

Madison, WI, USA

Ressler, Kerry

Professor

Emory University

Department of Psychiatry and Behavioral Sciences

Yerkes National Primate Research Center

Atlanta, GA, USA

Ruden, Douglas

Professor

Director of Epigenomics

Wayne State University

IEHS and OB/GYN

Detroit, MI, USA

Swanson, Penny

Professor

Environmental Physiology Program Manager

Environmental and Fisheries Sciences Division

Northwest Fisheries Science Center

NOAA-Fisheries

Seattle, WA, USA

Tonellato, Peter

Professor

University of Wisconsin Milwaukee

School of Public Health

Milwaukee, WI, USA

vom Saal, Frederick

Curators’ Professor

University of Missouri-Columbia

Division of Biological Sciences

Columbia, MO, USA

## Editorial Review Board

Bhandari, Ramji

Assistant Professor

Department of Biology

University of North Carolina Greensborough, USA

Breton, Carrie

Associate Professor

University Southern California, USA

Burghardt, Kyle J.

Assistant Professor of Pharmacy Practice

Eugene Applebaum College of Pharmacy and Health Sciences

Wayne State University

Detroit, MI, USA

Burris, Heather

Assistant Professor of Pediatrics

Perelman School of Medicine

University of Pennsylvania

Children's Hospital of Philadelphia, Neonatology

Philadelphia, PA, USA

Cardenas, Andres

Assistant Professor

University of California, Berkeley

Berkeley, CA, USA

Chen, Jia

Professor

Ichan School of Medicine at Mount Sinai

New York, NY, USA

Colacino, Justin

Assistant Professor

Environmental Health Sciences

University of Michigan School of Public Health, USA

Colicino, Elena

Assistant Professor

Environmental Medicine & Public Health

Icahn School of Medicine at Mount Sinai, USA

Cropley, Jennifer

ARC DECRA Fellow

Victor Chang Cardiac Research Institute

Australia

Davie, James

Department of Biochemistry and Medical Genetics

University of Manitoba

Winnipeg, MB, Canada

Dias, Brian G.

Assistant Professor

Emory University

Atlanta, GA, USA

Dinger, Marcel

Associate Professor

Garvan Institute of Medical Research

University of New South Wales, Australia Sydney

Eirin-Lopez, Jose M.

Assistant Professor

Department of Biological Sciences

Florida International University

Miami, FL, USA

Faulk, Christopher

Assistant Professor-Functional Genomics

University of Minnesota

AnSc/VetMed

St. Paul, MN, USA

Fry, Rebecca

Associate Professor

University of North Carolina

Environ Sci Engineering – Ops

Chapel Hill, NC, USA

Golding, Michael

Assistant Professor

Vet Med - Physiology & Pharmacology

Texas A&M University

College Station, TX, USA

Goodrich, Jaclyn

Research Assistant Professor

Environmental Health Sciences

University of Michigan

Ann Arbor, MI, USA

Greer, Eric Lieberman

Assistant Professor

Department of Pediatrics

Harvard Medical School Division of Newborn Medicine

Boston, MA, USA

Guerrero-Bosagna, Carlos

Avian Behavioural Genomics and Physiology Group

IFM Biology

Linköping University

Linköping, Sweden

Hostetler, Caroline

Postdoctoral Fellow

Oregon Health & Science University

Portland, OR, USA

Houghton, Franchesca

Associate Professor

Faculty of Medicine

University of Southampton

Southampton General Hospital, UK

Ideraabdullah, Folami

Assistant Professor

Departments of Genetics and Nutrition

University of North Carolina

Chapel Hill, NC, USA

Just, Allan

Assistant Professor

Environmental Medicine and Public Health

Division of Environmental Health

Icahn School of Medicine at Mount Sinai

New York, NY, USA

Kelsey, Gavin

Principal Research Scientist

Epigenetics Programme

The Babraham Institute

Cambridge, UK

Kile, Molly

Assistant Professor

Environmental & Occupational Health

College of Public Health and Human Sciences

Oregon State University

Corvallis, OR, USA

Kimmins, Sarah

Associate Professor, Associate Director,

McGill Centre for the Study of Reproduction

Departments of Animal Science, and Pharmacology and Therapeutics

McGill University

Montreal, QC, Canada

Kinnally, Erin

University of California – Davis

Psychology Department

Davis, CA, USA

Kotaja, Noora

Institute of Biomedicine

University of Turku

Turku, Finland

Kovalchuk, Igor

Professor, Board of Governors Research Chair in Epigenetics

Department of Biological Sciences

University of Lethbridge, Canada

Kovalchuk, Olga

Department of Biological Sciences

Professor and Board of Governors’ Research Chair

CIHR Chair in Gender and Health

University of Lethbridge, AB, Canada

Kramer, Jamie

Assistant Professor

Department of Biology

Western University

London, ON, Canada

Laiosa, Michael

Associate Professor

Joseph J. Zilber School of Public Health

University of Wisconsin-Milwaukee

Milwaukee, WI, USA

Lefebvre, Louis

Associate Professor

Department of Medical Genetics

University of British Columbia

Vancouver, BC, Canada

Marsit, Carmen

Professor

Department of Environmental Health

Department of Epidemiology

Director

HERCULES Exposome Research Center

Rollins School of Public Health

Emory University

Atlanta, GA, USA

McCullough, Shaun D.

U.S. Environmental Protection Agency

Research Biologist

National Health and Environmental Effects Research Laboratory

Environmental Public Health Division

Chapel Hill, NC, USA

Medici, Valentina

Associate Professor

University of California Davis

Department of Internal Medicine

Division of Gastroenterology and Hepatology

Sacramento, CA, USA

Meyer, Ralph G.

Associate Professor

Utah State University

School of Veterinary Medicine (WIMU)

College of Agricultural and Applied Sciences

Department of Animal, Dairy and Veterinary Sciences (ADVS)

Logan, UT, USA

Miska, Eric

Gurdon Institute

University of Cambridge, UK

Morison, Ian

Professor

Department of Pathology

University of Otago, New Zealand

Dunedin

Murphy, Susan K.

Associate Professor

Obstetrics and Gynecology

Duke University Medical Center

Durham, NC, USA

Nagel, Susan C.

Associate Professor

Obstetrics, Gynecology and Women’s Health

University of Missouri-Columbia

Columbia, MO, USA

Nakagawa, Shinichi

Professor

School of BEES

University of New South Wales

Sydney, Australia

Ng, Jane

Department of Pediatrics

University of Calgary, Canada

Nilsson, Eric

Research Professor

Washington State University

School of Biological Sciences

Pullman, WA, USA

Olson, David

Department of Obstetrics and Gynecology

University of Alberta, Canada

Öst, Anita

Principal Research Engineer

Linköping University

Department of Clinical and Experimental Medicine

Linköping

Sweden

Owens, Julie

Deputy Vice-Chancellor, Research

Deakin University

Geelong Waurn Ponds Campus

Victoria, Australia

Rassoulzadegan, Minoo

Inserm

University Nice

Sophia-Antipolis

Nice, France

Rivera, Rocio

Associate Professor

University of Missouri

Animal Science Research Center

Division of Animal Sciences

Columbia, MO, USA

Robert, Claude

Professeur Titulaire

Faculté des sciences de L'agriculture et de L'alimentation

Laval Université

Sces Agriculture et Alimentation-Dép. des Sciences Animales, Canada

Roth, Tania

Associate Professor

University of Delaware

Department of Psychological and Brain Sciences

Newark, DE, USA

Saffery, Richard

Group Leader

Murdoch Childrens Research Institute

Cancer & Disease Epigenetics Group

Melbourne, VIC, Australia

Saha, Ramendra

Assistant Professor

University of California, Merced

School of Natural Sciences, USA

Schmidt, Rebecca

Assistant Professor

Department of Public Health Sciences

University of California at Davis

MIND Institute Davis, CA, USA

Sharma, Abhay

Senior Principal Scientist

CSIR-Institute of Genomics and Integrative Biology

Council of Scientific and Industrial Research

South Campus

New Delhi

Skaar, David

Research Asst Professor, Toxicology

Center for Human Health and the Environment

Department of Biological Sciences

North Carolina State University

Raleigh, NC, USA

Stolzenberg, Danielle

Assistant Professor

Psychology Department

University of California at Davis

Davis, CA, USA

Sultan, Sonia

Professor

Department of Biology and Environmental Studies Program

Wesleyan University

Middletown, CT, USA

Suter, Catherine

Professor

Victor Chang Cardiac research Institute

Australia

Trasler, Jacquetta

Professor

McGill University Health Centre,

Montreal, QC, Canada

Watson, Erica

University Lecturer in Reproductive Biology

School of the Biological Sciences

University of Cambridge, UK

Youngson, Neil

Research Associate. PhD

School of Medical Sciences

The University of New South Wales

Sydney, NSW, Australia

Zama, Aparna

Assistant Research Professor

Department of Animal Sciences

Rutgers

New Brunswick, NJ, USA

Zeh, David W.

Professor of Biology

Vice Provost, Graduate Education

Dean, Graduate School

University of Nevada, Reno

Reno, NV, USA

Zhang, Xiaoyu

Assistant Professor

Department of Plant Biology

The University of Georgia

Athens, GA, USA

Zota, Ami

Assistant Professor

George Washington University

Environmental and Occupational Health

Washington, DC, USA

